# Comparison of Magnetic Auriculotherapy, Laser Auriculotherapy and Their Combination for Treatment of Insomnia in the Elderly: A Double-Blinded Randomised Trial

**DOI:** 10.1155/2019/3651268

**Published:** 2019-05-21

**Authors:** Lorna K. P. Suen, A. Molassiotis, S. K. W. Yueng, C. H. Yeh

**Affiliations:** ^1^School of Nursing, The Hong Kong Polytechnic University, Hung Hom, Hong Kong; ^2^School of Nursing, Johns Hopkins University, 525 N. Wolfe St., Room 421, Baltimore, MD 21205, USA

## Abstract

**Background:**

Insomnia is common amongst the elderly. With the adverse effects of prolonged use of hypnotics, the exploration of noninvasive and nonpharmacological complementary methods for insomnia is warranted. Auriculotherapy (AT) is a therapeutic approach where specific points on the auricle are stimulated to manage various physiological disorders. The purpose of this study is to determine the desirable treatment modality using AT to improve the sleep conditions of the elderly.

**Methods:**

A three-arm double-blinded randomised trial was conducted on 145 eligible subjects. This study investigated three minimally invasive procedures, namely, laser auriculotherapy (LAT), magneto-auriculotherapy (MAT), and their combination. Seven auricular points were used. Treatment was performed three times a week, for six weeks. Subjects were assessed at baseline, six weeks, and follow-up after six weeks, three months, and six months. Generalised estimating equations were used to evaluate interactions amongst the groups over time based on the Pittsburgh Sleep Quality Index (PSQI), sleep parameters using actigraphic monitoring, health-related quality of life (HRQOL) using SF-12, and PHQ-9 for depression status.

**Results:**

The treatment effects of the three procedures were comparable. Significant improvements were found in all of the subjective measures (PSQI, HRQOL, and PHQ-9) for individual groups over time. Improvements in the objective sleep parameters using actigraphic monitoring were detected in subjects who received MAT procedures but not in those who received LAT. The combined MAT and LAT approach did not show any advantage over MAT.

**Conclusions:**

The treatment effects of the three procedures were comparable in subjective parameters but not by objective measures using actigraphic monitoring. Longer therapeutic course and more frequent administration of LAT may be considered in future trials to achieve the optimal treatment effect.

**Trial Registration:**

This trial is registered with ClinicalTrials.gov: NCT02970695, registered May 2016.

## 1. Background

Insomnia is common amongst the elderly, and in some countries, the reported prevalence rate is over 60% [1]. Elderly people have difficulty falling asleep and maintaining sleep due to frequent awakenings [1]. Sleep loss in the aging population is associated with depression, anxiety, increased suicidal risks, comorbid chronic conditions, and high frequency of accidents and falls [2–4]. Moreover, chronic sleep disturbance can seriously compromise the overall quality of life of those who suffer from it [5, 6]. Given the adverse effects of prolonged use of hypnotics, such as morning sedation, impaired balance, drug dependence, depression, and amnesia [1, 7], the exploration of noninvasive and nonpharmacological complementary methods for insomnia amongst the elderly is warranted.

Auriculotherapy (AT) is a traditional Chinese medicine (TCM) approach in which the ear is viewed as a microsystem of the body [8]. AT is a therapeutic method where specific points on the auricle are stimulated to treat various bodily disorders. Different materials, such as acupuncture needles, press tack needles, seeds, magnetic pellets, or low-energy laser, could be applied on auricular points (denoted as “acupoints” in this paper) located on the external ear for therapeutic effect [8–11]. However, auricular acupuncture may induce patients' discomfort and cause infection and inflammation in the puncture sites. Magneto-auriculotherapy (MAT) has gradually emerged as a popular intervention for treating many chronic problems, such as insomnia [12], low back pain [13], constipation [14], and hypertension [15]. The effectiveness of magnetic pellets may be attributed to the functional changes caused by the interaction of magnetic fields with biological tissues. Such changes may be related to moving ions in blood [16].

Laser auriculotherapy (LAT) has also been widely used in different medical conditions, including insomnia [17], pain relief [18], and weight reduction [19]. The combination of LAT and other treatments proposed in the literature produces a synergistic effect. LAT has been combined with ear point pressing to treat bed wetting in children [20] and with auricular pressing therapy for alcoholic addiction [21]. According to TCM, the laser beam irradiates and stimulates the acupoint and activates the therapeutic effects of* qi* (energy flow), thereby regulating the functions of* zang-fu* (internal organs) and restoring* yin-yang *(equilibrium) to produce a therapeutic effect [22].

Laser treatment is noninvasive, painless and presents no risk of infection or cross infection [22]. As such, the therapeutic benefits of laser combined with MAT merit further investigation. LAT followed by MAT optimises the therapeutic effect because the latter allows continuous stimulation of acupoints after the laser treatment, as long as the magnet pellets on the ears are in situ.

In this study, three minimally invasive procedures, namely, LAT, MAT, and their combination, were investigated to determine the desirable treatment modality using AT to improve the sleep conditions of the elderly. Compared with the separate treatment procedures of MAT and LAT, their combined used is hypothesised to be more effective in improving the sleep conditions and thereby the quality of life of the elderly with insomnia.

## 2. Methods

### 2.1. Design

This study employed a three-arm double-blinded randomised trial. Eligible subjects were randomly divided into three groups by using a computer-generated randomised table and the equal proportion rule (1:1:1). The random coding was concealed from the subjects and evaluator by using opaque envelopes.

#### 2.1.1. Settings and Participants

Through convenience sampling, subjects were recruited from elderly centres in Hong Kong. A recruitment talk on AT was given to potential subjects in the targeted elderly centres. The definition of insomnia is adapted from existing literature [23]. After a preliminary screening, volunteers aged 65 years or above were recruited if they have the following symptoms: (1) difficulty falling or staying asleep and/or frequent nocturnal awakenings at least three nights per week, (2) sleep disturbance lasting for a minimum of 6 months, and (3) poor quality of sleep as indicated by a PSQI score greater than five. All the subjects fulfilled the criteria stipulated for the diagnosis of insomnia in the ‘Diagnostic and Statistical Manual of Mental Disorders', fifth edition [24]. The exclusion criteria were as follows: (1) presence of profound physical illnesses such as stroke, (2) diagnosis of obstructive sleep apnoea, (3) wearing a hearing aid or pacemaker in situ (to prevent the magnetic pellets from interfering with the devices), (4) received AT within the preceding six months, (5) suffering from aural injuries or infections, and (6) inability to understand instructions or provide consent.

### 2.2. Intervention and Procedures

#### 2.2.1. Acupoints Selection

**Figure 1:**
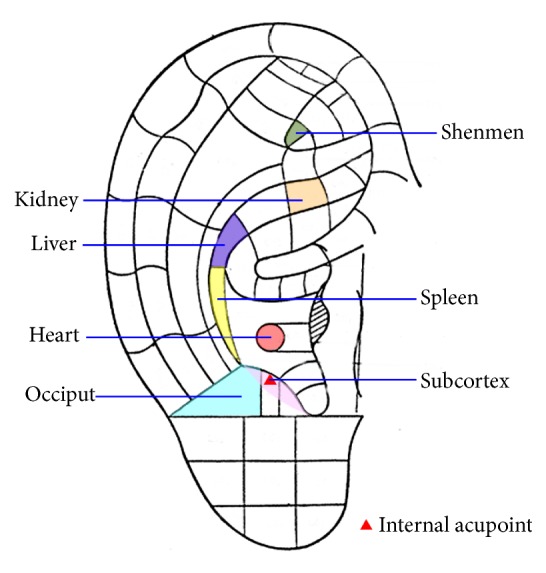
Selected auricular points for insomnia.

Seven auricular points, namely, “shenmen”, “heart”, “liver”, “spleen”, “kidney”, “occiput”, and “subcortex” (Figure 1), were selected because they promote sleep, as verified in a previous study by the first author [12]. The selection was based on the nomenclature and location of acupoints published by the China Standardisation Organising Committee (GB/T 13734-2008) [25]. Therapy was delivered by research personnel (SY) who had received intensive coaching from the first author (LS). Establishing the interrater reliability and accuracy of the ear point identification scheme ensured the fidelity of the study.

#### 2.2.2. Groupings

**Figure 2:**
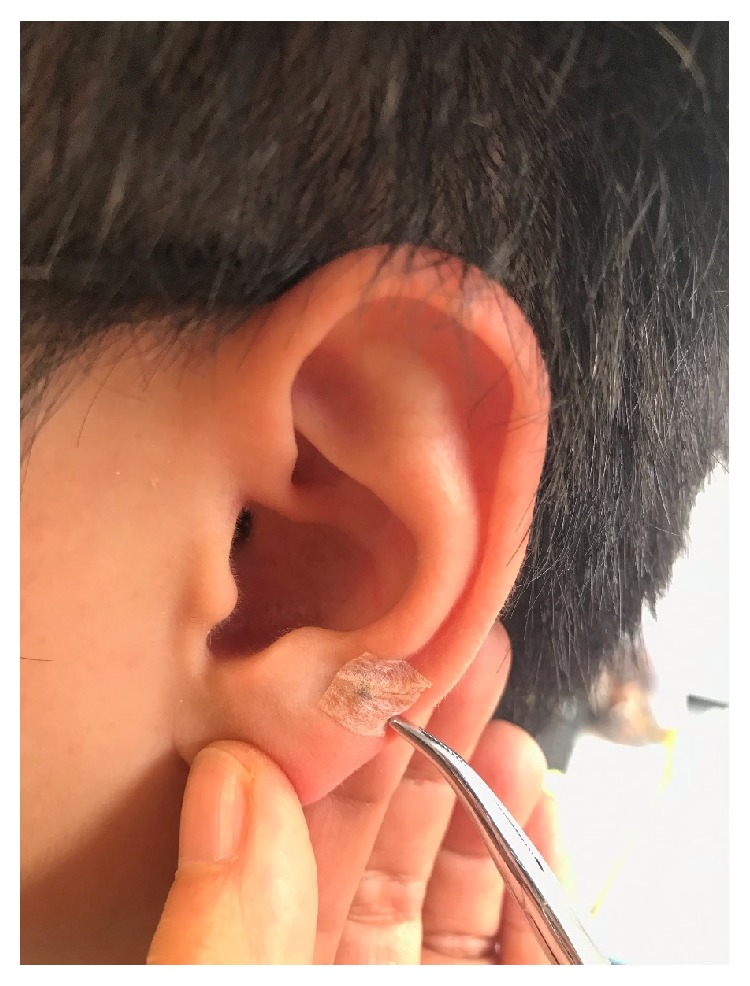
Administration of magneto-auriculotherapy.

*Group 1 (Placebo LAT and MAT)*. The laser device was switched to “power off” mode (i.e., deactivated) for acupoint ‘stimulation' to achieve blinding and the placebo effect, before the MAT. The subjects were asked to wear a pair of laser-protective goggles to ‘blind' them during treatment. MAT was then applied by placing magnetic pellets on the selected acupoints (Figure 2). Each magnetic pellet has an average gauss/pellet magnetic flux density of approximately 200 Gs (20 mT) and a diameter of 1.76 mm.

**Figure 3:**
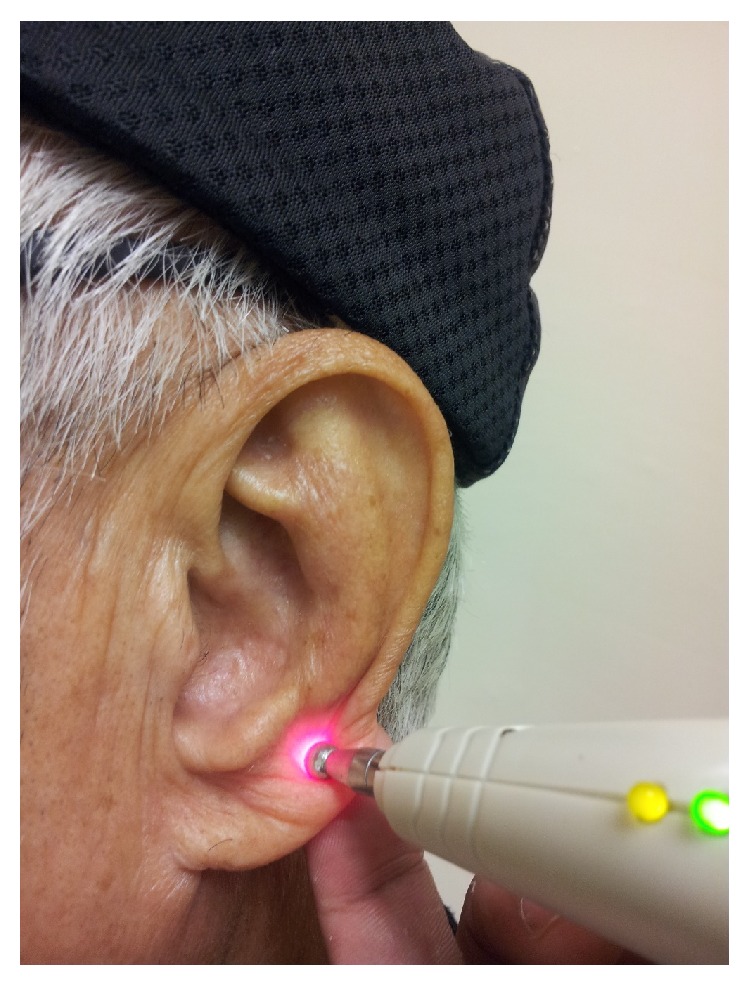
Administration of laser auriculotherapy.

*Group 2 (LAT and Placebo MAT)*. A laser device (Pointer Pulse™) was used for LAT. The device has a wavelength of 650 nm, average output power of 2.5 mW, energy density of 0.54 J/cm^2^ for 1 minute, and a pulse of 10 Hz, which is a commonly acceptable dosage for clinical use [18, 26]. LAT used low-level laser therapy (LLLT), in which the energy level emitted from the device is comparable with that of the teaching pointer. The continuous mode of the device was used to directly treat the acupoints for one minute (Figure 3). A plaster centred with a small dried stem of* Junci medulla*, a soft perennial plant, was provided to mimic MAT. In a previous study,* J. medulla *was successfully adopted as a placebo because it did not induce any physical pressure on the acupoints of the ear [12].

*Group 3 (Combined AT)*. The subjects received the combined LAT and MAT. The procedures for applying LAT and MAT were identical to the abovementioned descriptions.

#### 2.2.3. Procedures

Therapies were administered at elderly centres adjacent to the subjects' residences. The following procedures were standardised across the three groups to enhance the blinding effect. All therapies were administered in a room assigned for research purposes. Laser-protective goggles specific for the wavelengths of the laser device were provided to the subjects and researchers for eye protection.

The auricle of every subject was cleaned with 75% isopropyl alcohol before therapy. Only one ear was treated at a time. The treatment was applied alternately to the right ear in the first visit and then to the left ear in the subsequent visit. We replaced the experimental objects (i.e., magnetic pellets for true MAT or* J. medulla* for placebo MAT) every other day, that is, three times a week (except Sunday) to prevent local irritation of acupoints. The total treatment period was six weeks.

Participation in the study was voluntary. Written informed consent was obtained from each subject upon explaining the risks and benefits of their participation. Ethical approval was obtained from the Human Research Ethics Review Committee of the Hong Kong Polytechnic University. The study was conducted in accordance with the Declaration of Helsinki. Given their multiple visits to the centres to receive treatment, the subjects were provided a travel subsidy in the form of supermarket coupons upon completion of the study.

#### 2.2.4. Treatment Effect Evaluation

The subjects were assessed at baseline, at six weeks (postintervention), and during follow-up after six weeks, three months, and six months. To achieve evaluator blinding, the assessment was conducted by a different researcher who was unaware of the treatment modality given to the subjects. PSQI, which was used to collect data related to the sleep conditions of the subjects, was considered as the primary outcome. This instrument was scored from 0 to 21, and scores greater than five indicated poor sleep quality. Chong and Cheung [27] validated the Cantonese PSQI and reported a high internal consistency of 0.75.

The secondary outcomes considered are as follows: (1) actigraphic monitoring was conducted to collect sleep parameters, including sleep latency (minutes), waking after sleep onset (minutes), total sleep time (hours), and sleep efficiency (%). An Actiwatch' Spectrum Plus device with 0.025G ultra-high sensitivity and 32 Hz sampling rate was used in actigraphic monitoring. The subjects were requested to wear the device on the wrist of their nondominant hand 24 hours a day for 7 consecutive days to determine the overall sleep conditions within a certain period. Data were collected in epochs every 30 second. These epoch-by-epoch data were stored in the internal memory of the device until they could be downloaded to a computer. Actiware 6 Actigraph Analysis Software was used for sleep analysis. (2) The Chinese (HK) SF-12 v2©, an abbreviated version of the SF36 health questionnaire, was used to evaluate the health-related quality of life (HRQOL) of the subjects. This instrument covered 12 items, and the results were presented by a physical component score (PCS) and a mental component score (MCS). PCS and MCS ranged from 0 to 100, with higher scores indicating better HRQOL [28]. (3) Patient Health Questionnaire (PHQ-9) was also used. This instrument was validated as a useful tool for assessing depression status. The scores ranged from 0 to 27, and high scores indicated severe depression status [29]. The scale had a Cronbach's alpha of 0.82 and the recommended cut-off score was 8 [30].

The subjects' expectations and satisfaction towards the therapy were evaluated using a 10-point scale, with high scores indicating high expectations or satisfaction [31]. Data were likewise collected on sociodemographic characteristics including age, gender, marital status, educational level, religion, number of family members, body mass index, single/shared bed, comorbid illnesses, use of sleeping pills or aids, and current medications taken. Similarly monitored were the recruitment rate, compliance rate of the treatment protocol, and adverse effects arising from the therapy.

#### 2.2.5. Data Analyses

Descriptive statistics were determined on the sociodemographic and clinical characteristics of the subjects. The estimated mean and standard error were computed for the outcome variables of each time point. Association amongst categorical variables was estimated using x^2^ test or Fisher's exact test, where appropriate, to identify significant variables for inclusion in the generalised estimating equations (GEE) for adjustment. One-way analysis of variance was used to examine group differences. Primary analysis was conducted using GEE model with an autoregression correlation structure to evaluate interactions amongst the groups over time (baseline to six months follow-up) on the primary outcome (i.e., PSQI score) and secondary outcomes (sleep parameters using 24-hour actigraphic monitoring, quality of life using SF-12, and PHQ-9). Missing data were handled using GEE and assumed to be random [32]. The main analysis was repeated at postintervention and during the follow-up sessions (up to six months) for sensitivity analysis. SPSS version 25.0 (IBM Corporation, USA) was used for all statistical analyses. All statistical tests were two sided with significance level set at 0.05.

## 3. Results

The study was conducted from May 2016 to May 2018. Data were collected from 11 centres for the elderly, and the recruitment rate was 88.6%. A total of 147 eligible subjects were randomly divided into three groups (Group 1=50, Group 2=46, and Group 3=51).

### 3.1. Participants' Characteristics

The recruited subjects had an average age of 75.29 years± 6.99, with a mean duration of insomnia for 10.12 ± 10.67 years. Majority of the subjects (70.0%) did not take any medication to manage their sleep problems. The groups were essentially comparable and well balanced in terms of sociodemographic variables, including gender distribution, body mass index, education level, marital status, comorbid illnesses, and regular medications taken. However, age showed slight significant differences amongst the groups, and thus this variable was adjusted in the GEE models in subsequent analyses. According to the 24-hour actigraphic recordings, the subjects had an average of poor sleep quality (PSQI 12.63 ±3.24, sleep efficiency 72.33% ±16.09%), long sleep latency (27.03 ± 23.13 minutes), short total sleep time (3.76 ± 1.96 hours), and waking after sleep onset (90.31 ± 88.53 minutes). The subjects also had mild depression (PHQ-9 9.47 ± 6.07) and low HRQOL in terms of physical component (41.39 ± 8.51) and mental component (46.68 ± 12.34) (Table 1).

### 3.2. Compliance, Expectation, and Satisfaction towards the Treatment

**Figure 4:**
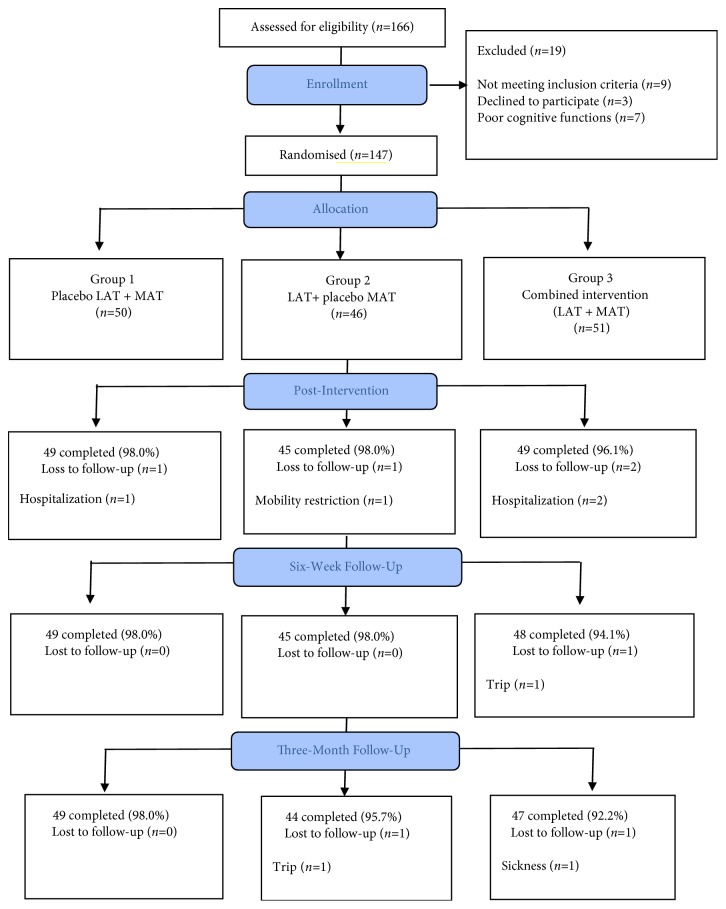
Flowchart of recruitment.

Compliance with the intervention protocol was high, at an average of 95.2% (*n *= 140) of the subjects continued with postintervention and all follow-up measurements until six months. The recruitment flowchart is illustrated in Figure 4. Although majority of the subjects (65.3%) had never tried complementary and alternative treatments, they generally exhibited strong confidence in the proposed therapy (7.82 of 10) and had a relatively high expectation of its effectiveness (7.73 of 10) before the trial. After the intervention, the subjects in Group 1 had the highest satisfaction from the therapy (7.86), followed by those in Group 3 (7.58) and Group 2 (7.02). A correlation analysis was conducted between expectations of the treatment effect and sleep parameters (PSQI, SE). However, no significant relationship was detected (p>0.05). Over 75% of the subjects (*n* = 109) indicated that they would definitely recommend the therapy to others. No specific adverse effects were observed arising from the therapy, apart from 16 cases (10.9%) who reported having mild skin irritation on the ears due to the adhesive tapes that were used to hold the experimental tools in place and 20 cases (13.6%) who felt tenderness on the acupoints (most of these subjects received MAT). The number of subjects who believed that they might be receiving placebo treatment was higher in Group 1 than in the other groups, although majority of the subjects (90%) believed that they were not receiving placebo (Table 2)

### 3.3. Treatment Effect

**Figure 5:**
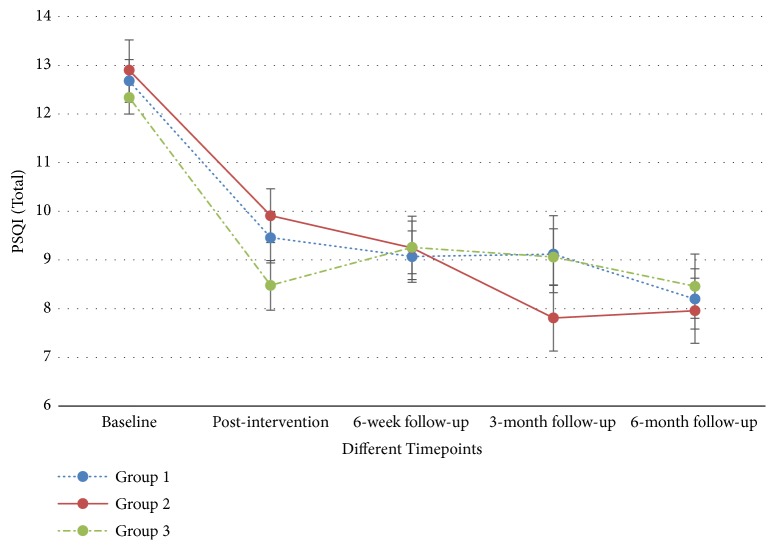
Pittsburgh Sleep Quality Index (PSQI, 0 to 21) across time.

**Figure 6:**
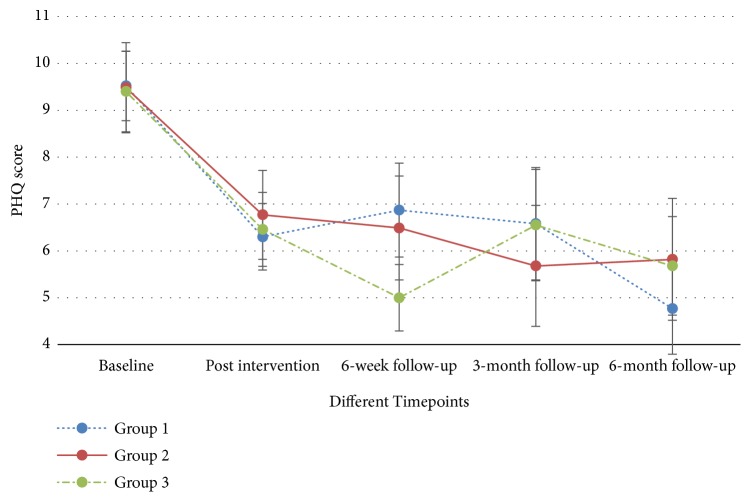
Patient Health Questionnaire (PHQ-9, 0 to 27) across time.

The differences in the primary and secondary outcomes of the three groups across different time points were compared through GEE model analysis, with adjustment for age. In general, no significant differences were detected in the outcomes (including PSQI, sleep parameters measured by actigraphic monitoring, SF-12, and PHQ-9) of the three groups (Table 3). However, significant differences were found in all of the subjective measures, including PSQI (Figure 5), SF-12 (physical and/or mental components) (Figures S1 and S2), and PHQ-9 (Figure 6) for individual groups over time. When the sleep conditions were evaluated by actigraphic monitoring, significant differences in ‘waking after sleep onset' (minutes) (Figure S3) and ‘sleep efficiency' (%) (Figure S4) were detected only in Groups 1 and 3 (Table 4). The completers' analysis showed consistent findings on the primary and secondary outcomes of the trial.

## 4. Discussion

Numerous studies that used AT to manage sleep problems in China encountered methodological flaws, which rendered their findings unconvincing. The common problems included lack of details on how randomisation and allocation concealment were conducted, absence of objective measurements and a control or placebo group as well as failure to report the use of blinding and selective reporting of findings [33–35]. The present study was performed using a scientific approach to identify the optimum treatment protocol for AT in improving the sleep conditions and quality of life of the elderly with insomnia. This meticulous randomised controlled trial (RCT) could provide scientific evidence regarding causal relationships between interventions and outcomes.

In general, the treatment effect was comparable amongst the three AT protocols. However, significant improvements were observed in all of the subjective measures, including sleep conditions measured by PSQI, HRQOL, and depression status for individual groups over time. When the sleep conditions were evaluated by objective measures using actigraphic monitoring, significant differences in “waking after sleep onset” (minutes) and “sleep efficiency” (%) were only noted in Groups 1 and 3. The use of actigraphs has been widely recognised as an objective measurement that could provide longitudinal assessment of sleep patterns in a natural environment [36, 37]. This technique could similarly provide valid measures that may not be influenced by subject bias. Significant reduction in the awakening time after sleep onset and increase in sleep efficiency were only detected in subjects who received MAT protocols (i.e., Groups 1 and 3) but not in those who received only the LAT protocol. Despite the improved sleep efficiency of the subjects at postintervention and during the follow-up periods, the evaluated indices remained below 85%, a common cut-off percentage that indicates the presence of sleep disturbances [38]. Sleep efficiency is calculated by dividing the total sleep time by total bedtime; a higher sleep efficiency means better sleep quantity and quality. A long therapeutic course, such as 10–12 weeks, may be necessary to further elevate sleep efficiency to a desirable level through sustainable treatment effect.

MAT could provide continuous stimulation of acupoints as long as the magnetic pellets on the ears are in situ, and the subjects could receive laser stimulation to the acupoints on the day of treatment. The synergistic effect of the combined MAT and LAT was demonstrated in two previous trials [39, 40] conducted by the research team. In a double-blind RCT for osteoarthritic knee, the subjects who received the combined AT protocols exhibited stronger treatment effects in terms of pain relief, ambulation status, and range of knee movements compared with those treated with separate MAT or LAT [39], whereas in another double-blinded RCT for aging males with lower urinary tract symptoms, a combined AT protocol exhibited a stronger therapeutic effect in relieving voiding problems, improving the urinary flow rate, and minimizing the postvoid residual urine than the placebo group or MAT alone [40]. However the combined MAT and LAT approach did not show any advantage over the separate MAT protocol in current study. Therefore a greater frequency for LAT administration, such as daily application adopted in previous studies [41–44], may be considered in future studies to enhance the treatment effect and possibly improve its synergistic effect when combined with MAT.

Numerous clinical trials reported the use of MAT on different disorders, including but not limited to sleep disturbances [12, 45, 46], low back pain [13], constipation [14], and obesity [47]. The effectiveness of MAT may relate to the interaction of magnetic fields with blood flow and calcium channel proteins in the cell membrane. Such interactions may elicit functional body changes [16, 48, 49]. Meanwhile, LAT is a noninvasive alternative to needle acupuncture [22] and has been adopted to increase pain threshold [50] as well as relieve musculoskeletal pain [18] and insomnia [17]. The laser beam not only irradiates and stimulates acupoints but also triggers the energy flow (*qi*) and regulates the functions of internal organs to achieve a therapeutic effect [22].

According to neuroembryonic theory, Dr. Paul Nogier viewed the auricle as a homunculus of the human body and has a similar shape to an inverted foetus [8]. As such, appropriate stimulation of specific ear acupoints can achieve therapeutic effects [51]. In the present study, the selection of auricular acupoints was based on the TCM theory and ideas borrowed from modern medicine. For example, treating the “heart” can calm the mind, while soothing the “liver” could regulate the flow of* qi*, particularly when insomnia is caused by liver* qi* stagnation [52]. “Shenmen” and “occiput” are believed to tranquilise the mind, and the ‘subcortex' can harmonise cortex excitement and inhibition [12].

A population-based epidemiological study conducted on 5,000 subjects in Hong Kong reported that insomnia was highly prevalent amongst Chinese adults and was associated with poor mental status and quality of life [53]. The present study reported PCS scores of the subjects comparable with those of Hong Kong people with insomnia (43.21 in our study versus 41.39 population norm), but stated a slightly higher MCS (46.68 versus 36.36). HRQOL (in PCS and MCS) improved and depression declined over time in subjects treated with AT. Thus, quality of life and emotional status of the subjects may be positively associated with sleep improvement after the therapy.

No specific adverse effects were observed to arise from the therapy, apart from a small number of reported cases (10.9%) having mild skin irritation on the ears from the adhesive tapes used to hold the experimental tools in place. Moreover, 20 cases (13.6%) reported tenderness on the acupoints, and most of these subjects received MAT. According to the auricular diagnosis system, the areas of the auricle with heightened tenderness upon touching correspond to specific areas of the body where pathological conditions exist [54, 55]. Applying magnetic pellets may induce physical pressure on the ear acupoints and cause tenderness, especially in cases with disequilibrium of bodily functions (e.g., insomnia) corresponding to specific acupoints. The tenderness on the reflective acupoints experienced by the subjects may be considered part of the treatment process rather than adverse effects of AT.

The high compliance rate (95.2%) and positive impression of the therapy indirectly indicated that blinding was successful because of the successful placebo application in the trial. Over 75% of the participants expressed that they would definitely recommend the therapy to others. The findings of this trial can provide valuable information and increase understanding of the therapeutic effect of AT, whether separate or combined MAT and LAT. Longer therapy duration may be considered in future trials to determine further improvements in the outcome variables. The proposed treatment approach can be considered as a noninvasive strategy for managing insomnia amongst the elderly.

Although actigraphic monitoring is a reliable objective measure in sleep study, changes in different sleep stages caused by the therapy cannot be ascertained. The recruited elders generally obtained low education levels and had a mean age of over 75, and therefore, using a sleep log to verify the actigraphic data was not feasible. Night-to-night variability in the sleeping patterns of subjects may also be affected by physical, psychological, and/or environmental factors. Due to the above limitations, the actigraphic data must be interpreted with caution. The exact mechanisms remain unknown regarding the interaction of magnetic fields with biological tissues, which results in functional changes. Further studies from the biomedical perspective are required to elucidate the biological pathway of the treatment and effect, such as to examine the impact of the treatment protocols on the changes in sleep biomarkers when sleep prosperity is achieved.

## 5. Implications

This study provides valuable findings regarding the therapeutic effect of different protocols using MAT, LAT, or their combination. In general, the AT protocols under testing may be considered as a noninvasive approach with minimal adverse effects for managing sleep problems amongst the elderly. The findings of this study provide important implications to guide future research and apply evidence-based practice in the community via service provision to manage this common problem.

## 6. Conclusion

The treatment effects of the three protocols were comparable in terms of self-reported sleep conditions, HRQOL, and depression status. In several parameters, such therapeutic effects may be sustained at six-month follow-up. Significant improvement in the objective sleep parameters could be observed in subjects who received MAT protocols but not in those who received LAT. The combined MAT and LAT approach did not show any advantage over MAT. Longer therapeutic course and more frequent administration of LAT may be considered in future trials to achieve the optimal treatment effect. In general, the proposed AT protocols were demonstrated to be a well-received treatment modality with minimal adverse effects and effectively improved sleep conditions of the elderly with insomnia.

## Figures and Tables

**Table 1:** Sociodemographic and baseline characteristics of the participants sample (*N*=147).

	All (*N*=147)	Group 1 Placebo LAT & MAT (*n*=50)	Group 2 LAT & placebo MAT (*n*=46)	Group 3 Combined AT (*n*=51)	*P*-value
Age (years)					❖0.025*∗*
Mean (SD)	75.29 (6.99)	76.02 (7.02)	76.80 (6.16)	73.20 (7.29)	

Gender					
Male	19	7	5	7	★ 0.907
Female	128	43	41	44	

Education level					
Primary or below	100	32	35	33	*◈*0.734
Secondary	39	15	9	15	
Tertiary or above	8	3	2	3	

Marital status					
Single	5	2	1	2	*◈*0.971
Married	82	29	26	27	
Divorced/Widowed	60	19	19	22	

Religion					
No	75	26	22	27	★ 0.895
Yes	72	24	24	24	

Body mass index (kg/m^2^)	22.29 (3.73)	22.85 (3.48)	21.50 (3.57)	22.46 (4.04)	❖ 0.076

Shared bed					
No	109	33	38	38	★ 0.188
Yes	38	17	8	13	

Living alone					
No	66	20	27	19	★ 0.079
Yes	81	30	19	32	

Sleeping pills taken					
No	103	34	33	36	*◈*0.391
Previous user	32	14	7	11	
Current user	12	2	6	4	

Duration of insomnia (years) Mean (SD)	10.12 (10.67)	9.23 (8.34)	11.15 (13.09)	10.06 (10.40)	❖ 0.678

Comorbid illness					
No	22	5	5	12	★ 0.488
Yes	125	45	41	39	

Regular drugs taken					
No	32	10	7	15	★ 0.441
Yes	115	40	39	36	

PSQI (total) (0-21)	12.63 (3.24)	12.67 (3.15)	12.89 (4.13)	12.35 (2.34)	❖ 0.715

Sleep latency (min)	27.03 (23.13)	26.56 (23.76)	28.68 (24.86)	26.00 (21.19)	❖ 0.839

Total sleep time (hours)	3.76 (1.96)	3.93 (1.93)	3.74 (1.86)	3.62 (2.10)	❖ 0.734

Wake after sleep onset (minutes)	90.31 (88.53)	92.06 (91.44)	90.03 (99.56)	88.86 (75.95)	❖ 0.984

Sleep efficiency (%)	72.33 (16.09)	72.44 (15.91)	74.25 (16.04)	70.50 (16.42)	❖ 0.521

SF-12 (PCS)	41.39 (8.51)	40.65 (7.58)	41.43 (9.69)	42.07 (8.36)	❖ 0.707

SF-12 (MCS)	46.68 (12.34)	48.22 (11.76)	46.19 (13.49)	45.62 (11.89)	❖ 0.542

PHQ-9	9.47 (6.07)	9.54 (5.34)	9.52 (6.69)	9.35 (6.26)	❖ 0.986

SD, standard deviation.

PSQI: Pittsburgh sleep quality index.

SF-12 (PCS): physical component score.

SF-12 (MCS): mental component score.

PHQ-9: Patient Health Questionnaire, for depression.

❖One-way analysis of variance.

*◈*Fisher's exact test.

★Chi-square test.

*∗*Statistically significant at *P* < 0.05.

**Table 2:** Reported adverse effects, expectations, and satisfaction towards the therapy ¥.

	All (*n*=147)	Group 1 Placebo LAT & MAT (*n*=50)	Group 2 LAT & placebo MAT (*n*=46)	Group 3 Combined AT (*n*=51)	*P-*value
Have you used complementary therapies in the past?#					★ 0.423
No	96 (65.3%)	33	33	30	
Yes	51 (34.7%)	17	13	21	

How much faith do you have in complementary therapies in general (0 to 10) #	7.82 (2.13)	8.40 (1.94)	7.48 (2.18)	7.55 (2.18)	❖ 0.056

Expectation for treatment effect towards MAT (0 to 10) #	7.41 (2.10)	7.96 (2.11)	6.93 (2.03)	7.31 (2.07)	❖ 0.051

Expectation for treatment effect towards LAT (0 to 10) #	7.41 (2.13)	8.08 (2.00)	6.87 (2.09)	7.25 (2.15)	❖ 0.016*∗*

Average expectation for treatment effect (0 to 10) #	7.73 (2.13)	8.40 (2.03)	7.20 (2.03)	7.57 (2.18)	❖ 0.016*∗*

Ear itchiness ¥	16 (10.9%)	8*∗* (resolve automatically)	2*∗* (resolve automatically)	6*∗* (resolve automatically)	- - -

Tenderness on acupoints ¥	20 (13.6%)	9*∗* (resolve automatically)	1*∗* (resolve automatically)	10*∗* (resolve automatically)	- - -

Satisfaction towards therapy (0 to 10) ¥	7.58 (2.37)	7.86 (2.03)	7.02 (2.68)	7.58 (2.37)	❖ 0.225

Thought that they might be receiving placebo treatment ¥					
No	126 (90.0%)	39	41	46	*◈*0.011*∗*
Yes	14 (10.0%)	10	3	1	

Will recommend this therapy to others ¥					*◈* 0.166
Definitely will	109 (75.7%)	38	29	42	
Maybe	20 (13.9%)	5	9	6	
No	15 (10.4%)	6	7	2	

Mean (standard deviation) unless otherwise noted.

*Ω*Association between variables was determined by chi-square analyses or Fisher's-exact test where appropriate.

#Evaluated before the intervention.

¥Evaluated after the intervention has been completed.

*∗*“Certain” causality.

❖One-way analysis of variance.

*◈*Fisher's exact test.

★Chi-square test.

*∗*Statistically significant at *P*<0.05.

**Table 3:** Outcome variables across three groups at different timepoints.

				Pairwise comparisons between groups *▾*		
	Group 1 Placebo LAT & MAT (*n*=50)	Group 2 LAT & placebo MAT (*n*=46)	Group 3 Combined AT (*n*=51)	Group 1 vs Group 2	Group 1 vs Group 3	Group 3 vs Group 2
	Estimated mean (SE) *※*	Estimated mean (SE) *※*	Estimated mean (SE) *※*	*β* (95% CI)	*β* (95% CI)	*β* (95% CI)
*PSQI (total) (0-21)*						
Baseline	12.68 (0.44)	12.90 (0.62)	12.34 (0.34)			
Post intervention	9.46 (0.52)	9.91 (0.55)	8.48 (0.51)	0.23 (-1.44, 1.89)	-0.65 (-2.36, 1.06)	0.87 (-0.55, 2.30)
6 weeks follow up	9.07 (0.53)	9.25 (0.65)	9.26 (0.54)	-0.50 (-2.07, 1.97)	0.53 (-1.14, 2.19)	-0.58 (-2.54, 1.38)
3 months follow up	9.12 (0.79)	7.81 (0.68)	9.06 (0.58)	-1.54 (-4.00, 0.93)	0.27 (-1.88, 2.42)	-1.81 (-3.99, 0.37)
6-month follow up	8.20 (0.62)	7.96 (0.67)	8.46 (0.66)	-0.47 (-2.68, 1.75)	0.59 (-1.38, 2.56)	-1.06 (-3.16, 1.05)

*Sleep latency (minutes)*						
Baseline	26.82 (3.33)	29.16 (3.59)	25.17 (2.86)			
Post intervention	25.29 (2.97)	25.00 (3.32)	26.17 (3.03)	(-13.29, 8.02)	2.23 (-8.11, 12.57)	-4.86 (-15.33, 5.60)
6 weeks follow up	19.35 (3.22)	26.56 (3.39)	23.68 (3.30)	4.87 (-7.73, 17.47)	5.67 (-5.89, 17.23)	-0.80 (-11.77, 10.18)
3 months follow up	17.99 (3.37)	21.29 (3.56)	23.90 (4.22)	0.96 (-11.49, 13.41)	7.25 (-6.93, 21.43)	-6.29 (-20.65, 8.07)
6-month follow up	19.66 (5.31)	23.42 (5.39)	19.07 (4.34)	1.43 (-15.79, 18.64)	0.76 (-15.34, 16.86)	0.67 (-15.85, 17.18)

*Total sleep time (hours) *						
Baseline	3.93 (0.27)	3.75 (0.28)	3.61 (0.30)			
Post intervention	3.93 (2.49)	4.05 (0.30)	3.96 (0.29)	0.31 (-0.67, 1.28)	0.35 (-0.65, 1.36)	-0.05 (-1.17, 1.08)
6 weeks follow up	3.81 (0.27)	3.90 (0.29)	3.85 (0.25)	0.27 (0.68, 1.23)	0.36 (-0.59, 1.31)	-0.09 (-1.16, 0.99)
3 months follow up	3.66 (0.44)	4.26 (0.49)	3.38 (0.24)	0.77 (-0.81, 2.36)	0.034 (-1.32, 1.39)	0.74 (-0.61, 2.09)
6-month follow up	3.92 (0.49)	4.42 (0.61)	3.71 (0.45)	0.68 (-1.03, 2.38)	0.112 (-1.45, 1.68)	0.57 (-1.14, 2.27)

*Wake after sleep onset (minutes) *						
Baseline	92.32 (12.83)	90.51 (14.64)	88.33 (10.31)			
Post intervention	69.44 (10.41)	71.60 (9.49)	65.84 (8.86)	3.97 (-22.03, 29.96)	0.38 (-23.41, 24.18)	3.58 (-24.42, 31.59)
6 weeks follow up	51.45 (5.10)	61.40 (8.60)	62.34 (7.07)	11.76 (-24.44, 47.95)	14.88 (-17.49, 47.25)	-3.13 (-39.79, 33.54)
3 months follow up	50.82 (9.13)	74.74 (20.96)	63.90 (12.00)	25.73 (-38.06, 89.51)	17.06 (-30.43, 64.55)	8.67 (-55.72, 73.06)
6-month follow up	48.81 (9.66)	80.87 (26.46)	61.59 (11.04)	33.87 (-38.57, 106.30)	16.77 (-30.71, 64.24)	17.10 (-55.55, 89.75)

*Sleep efficiency (%) *						
Baseline	72.34 (2.23)	74.06 (2.38)	70.70 (2.23)			
Post intervention	78.28 (1.90)	76.94 (1.93)	78.86 (1.64 )	-3.06 (-8.52, 2.40)	2.22 (-4.06, 8.50)	-5.28 (-11.61, 1.05)
6 weeks follow up	77.57 (1.80)	76.14 (2.49)	74.57 (2.21)	-3.14 (-9.31, 3.03)	-1.36 (-7.71, 5.00)	-1.78 (-8.75, 5.18)
12-week follow up	81.32 (1.98)	78.02 (3.56)	78.82 (2.34)	-5.03 (-15.44, 5.39)	-0.87 (-10.27, 8.54)	-4.16 (-15.43, 7.10)
6-month follow up	76.02 (4.84)	77.37 (4.64)	80.19 (2.20)	-0.37 (-14.64, 13.90)	5.81 (-6.18, 17.80)	-6.18 (-18.97, 6.62)

*SF-12 (PCS)*						
Baseline	40.87 (1.09)	41.84 (1.37)	41.64 (1.19)	-		
Post intervention	44.46 (1.23)	43.12 (1.38)	42.96 (1.22)	2.31 (-6.00, 1.39)	-2.27 (-5.69, 1.14)	-0.035 (-3.62, 3.55)
6-weeks follow up	45.04 (1.34)	42.58 (1.44)	40.33 (1.51)	-3.42 (-7.51, 0.66)	-5.47 (-9.89, -1.06)*∗*	2.05 (-2.06, 6.15)
3 months follow up	45.55 (1.34)	46.21 (1.42)	41.30 (1.66)	-0.31 (-4.69, 4.07)	-5.02 (-9.53, -0.52)*∗*	4.72 (0.13, 9.30)*∗*
6-month follow up	46.26 (1.36)	45.96 (1.41)	41.99 (2.29)	-1.26 (-5.63, 3.11)	-5.03 (-10.55, 0.48)	3.77 (-2.06, 9.60)

*SF-12 (MCS)*						
Baseline	47.99 (1.63)	45.77 (1.95)	46.05 (1.63)			
Post intervention	51.84 (1.64)	47.35 (1.78)	50.58 (.1.37)	-2.26 (-7.10, 2.57)	0.675 (-4.02, 5.37)	-2.94 (-7.42, 1.54)
6 weeks follow up	50.97 (1.61)	50.89 (1.84)	53.15 (1.65)	2.14 (-2.62, 6.90)	4.12 (-0.95, 9.18)	-1.98 (-6.73, 2.77)
3 months follow up	48.05 (2.31)	51.31 (2.27)	50.55 (1.76)	5.48 (-1.52, 12.47)	4.44 (-1.80, 10.67)	1.04 (-5.38, 7.47)
6-month follow up	51.64 (2.00)	52.24 (2.05)	51.05 (2.20)	2.82 (-3.83, 9.47)	1.35 (-5.24, 7.94)	1.47 (-5.41, 8.36)

*PHQ-9 (0-27)*						
Baseline	9.52 (0.74)	9.48 (0.96)	9.40 (0.86)			
Post intervention	6.30 (0.71)	6.77 (0.95)	6.46 (0.79)	0.504 (-1.52, 2.52)	0.28 (-1.78, 2.35)	0.22 (-1.72, 2.17)
6 weeks follow up	6.87 (1.00)	6.49 (1.11)	5.00 (0.71)	-0.35 (-2.73, 2.04)	-1.75 (-4.29, 0.78)	1.41 (-0.64, 3.45)
3 months follow up	6.58 (1.20)	5.68 (1.29)	6.55 (1.19)	--0.87 (-3.93, 2.21)	0.09 (-3.08, 3.26)	-0.96 (-4.05, 2.14)
6-month follow up	4.77 (0.97)	5.82 (1.30)	5.68 (1.05)	1.10 (-1.88, 4.01)	1.04 (-1.87, 3.95)	0.06 (-3.07, 3.18)

*※*Estimated mean and standard error (SE) from generalized estimating equations.

PSQI: Pittsburgh sleep quality index.

SF-12 (PCS): physical component score.

SF-12 (MCS): mental component score.

PHQ-9: Patient Health Questionnaire, for depression.

*▾*Adjusted for age.

*∗*Statistically significant at *P*< 0.05.

**Table 4:** Change in different outcome variables over time for individual groups using generalized estimating equations.

	Post-hoc analyses *◈▾*							
	(1) vs (2)		(1) vs (3)		(1) vs (4)		(1) vs (5)	
	*β* (95% CI)	*p*-value	*β* (95% CI)	*p*-value	*β* (95% CI)	*p*-value	*β* (95% CI)	*p*-value
Group 1 Placebo LAT & MAT (*n*=50)								
PSQI (0-21, total)	-3.21 (-4.56, -1.87)	0.000*∗∗∗*	-3.61 (-4.83, -2.38)	0.000*∗∗∗*	-3.56 (-5.28, -1.83)	0.000*∗∗∗*	-4.47 (-5.95. -3.00)	0.000*∗∗∗*
Sleep latency (minutes)	-1.53 (-8.97, 5.92)	0.688	-7.47 (-16.74, 1.81)	0.115	-8.83 (-17.48, -0.18)	0.045*∗*	-7.16 (-19.08, 4.75)	0.239
Total sleep time (hours)	-0.01 (-0.59, 0.58)	0.989	-0.12 (-0.69, 0.45)	0.681	-0.27 (-1.39, 0.86)	0.642	-0.01 (-1.12, 1.09)	0.985
Wake after sleep onset (minutes)	-22.87 (-38.01, -7.74)	0.003*∗∗*	-40.87 (-63.38, -18.36)	0.000*∗∗∗*	-41.49 (-74,47, -8.52)	0.014*∗*	-43.51 (-77.26, -9.76)	0.012*∗*
Sleep efficiency (%)	5.94 (2.12, 9.76)	0.002*∗∗*	5.22 (1.36, 9.09)	0.008*∗∗*	8.98 (3.07, 14.90)	0.003*∗∗*	3.68 (-5.99, 13.35)	0.455
SF12 (PCS)	3.59 (1.09, 6.09)	0.005*∗∗*	4.16 (1.06, 7.27)	0.009*∗∗*	4.68 (1.64, 7.72)	0.003*∗∗*	5.38 (2.58, 8.19)	0.000*∗∗∗*
SF12 (MCS)	3.85 (0.29, 7.41)	0.034*∗*	2.98 (0.61, 6.57)	0.103	0.06 (-4.76, 4.88)	0.980	3.65 (-0.83, 8.12)	0.110
PHQ-9 (0-27)	-3.22 (-4.73, -1.71)	0.000*∗∗∗*	-2.65 (-4.63, -0.66)	0.009*∗∗*	-2.94 (-5.17, -0.71)	0.010*∗*	-4.75 (-6.69, -2.82)	0.000*∗∗∗*

Group 2 LAT & placebo MAT (*n*=46)								
PSQI (0-21, total)	-2.99 (-3.96, -2.02)	0.000*∗∗∗*	-3.66 (-5.26, -2.05)	0.000*∗∗∗*	-5.09 (-6.85, -3.33)	0.000*∗∗∗*	-4.94 (-6.60, -3.28)	0.000*∗∗∗*
Sleep latency (minutes)	-4.16 (-11.78, 3.46)	0.284	-2.59 (-11.15, 5.96)	0.552	-7.87 (-16.82, 1.08)	0.085	-5,74 (-18.18, 6.71)	0.366
Total sleep time (hours)	0.30 (-0.47, 1.08)	0.444	0.15 (-0.61, 0.92)	0.695	0.51 (-0.61, 1.62)	0.375	0.67 (-0.64, 1.97)	0.316
Wake after sleep onset (minutes)	-18.91 (-40.04, 2.23)	0.080	-29.11 (-57.49, -0.72)	0.044*∗*	-15.76 (-70.40, 38.88)	0.572	-9.64 (-74.21, 54.94)	0.770
Sleep efficiency (%)	2.88 (-1.03, 6.78)	0.148	2.08 (-2.72, 6.89)	0.396	3.96 (-4.62, 12.53)	0.366	3.31 (-7.32, 13.95)	0.542
SF12 (PCS)	1.28 (-1.44, 4.00)	0.357	0.74 (-1.91, 3.39)	0.585	4.37 (1.21, 7.53)	0.007*∗∗*	4.12 (0.75, 7.49)	0.017*∗*
SF12 (MCS)	1.58 (-1.68, 4.85)	0.341	5.12 (1.99, 8.25)	0.001*∗∗*	5.54 (0.48, 10.61)	0.032*∗*	6.47 (1.55, 11.39)	0.010*∗*
PHQ-9 (0-27)	-2.72 (-4.06, -1.38)	0.000*∗∗∗*	-2.99 (-4.31, -1.67)	0.000*∗∗∗*	-3.80 (-5.92, -1.69)	0.000*∗∗∗*	-3.66 (-5.91, -1.41)	0.001*∗∗*

Group 3 Combined AT (*n*=51)								
PSQI (0-21, total)	-3.86 (-4.91, -2.82)	0.000*∗∗∗*	-3.08 (-4.20, -1.95)	0.000*∗∗∗*	-3.28 (-4.57, -2.00)	0.000*∗∗∗*	-3.88 (-5.18. 2.58)	0.000*∗∗∗*
Sleep latency (minutes)	0.70 (-6.47, 7.88)	0.848	-1.80 (-8.70, 5.10)	0.610	-1.58 (-12.82, 9.67)	0.784	-6.40 (-17.34, 4.54)	0.251
Total sleep time (hours)	0.35 (-0.46, 1.16)	0.399	0.24 (-0.52, 1.00)	0.535	-0.23 (-0.99, 0.52)	0.543	0.10 (-1.00, 1.21)	0.858
Wake after sleep onset (minutes)	-22.49 (-40.86, -4.12)	0.016*∗*	-25.98 (-49.25, -2.72)	0.029*∗*	-24.43 (-58.62, 9.76)	0.161	-26.74 (-60.49, 7.01)	0.120
Sleep efficiency (%)	8.16 (1.18, 13.14)	0.001*∗∗*	3.87 (-1.18, 8.91)	0.133	8.12 (0.80, 15.44)	0.030*∗*	9.49 (2.31, 16,68)	0.010*∗*
SF12 (PCS)	1.32 (-1.01, 3.64)	0.268	-1.31 (-4.45, 1.82)	0.413	-0.35 (-3.67, 2.98)	0.839	0.35 (-4.40, 5.10)	0.886
SF12 (MCS)	4.52 (1.46, 7.59)	0.004*∗∗*	7.10 (3.52, 10.67)	0.000*∗∗∗*	4.50 (0.55, 8.45)	0.026*∗*	5.00 (0.18, 9.82)	0.042*∗*
PHQ-9 (0-27)	-2.94 (-4.35, -1.53)	0.000*∗∗∗*	-4.40 (-5.97, -2.83)	0.000*∗∗∗*	-2.85 (-5.10, -0.59)	0.013*∗*	-3.71 (-5.89, -1.54)	0.001*∗∗*

SE, standard error; CI, confidence interval.

*◈*Timepoints: (1) = baseline, (2) = postintervention, (3) = 6-week follow up, (4) = 3-month follow up, and (5) = 6-month follow-up.

*▾*Adjusted for age.

PSQI = Pittsburgh sleep quality index.

SF-12 (PCS): physical component score.

SF-12 (MCS): mental component score.

PHQ-9: Patient Health Questionnaire.

*∗*Statistically significant at *P*< 0.05.

*∗∗* Statistically significant at *P*<0.01.

*∗∗∗* Statistically significant at *P*<0.001.

## Data Availability

The datasets used and/or analysed during the current study are available from the corresponding author on reasonable request

## Supplementary Materials

Supplementary Materials Figure S1: wake after sleep onset (minutes) across time. Figure S2: sleep efficiency (%) across time. Figure S3: SF-12 (physical component) across time. Figure S4: SF-12 (mental component) across time.
